# Creatine Metabolism in Female Reproduction, Pregnancy and Newborn Health

**DOI:** 10.3390/nu13020490

**Published:** 2021-02-02

**Authors:** Anna Maria Muccini, Nhi T. Tran, Deborah L. de Guingand, Mamatha Philip, Paul A. Della Gatta, Robert Galinsky, Larry S. Sherman, Meredith A. Kelleher, Kirsten R. Palmer, Mary J. Berry, David W. Walker, Rod J. Snow, Stacey J. Ellery

**Affiliations:** 1 The Ritchie Centre, Hudson Institute of Medical Research, Clayton, VIC 3168, Australia; ammuc2@student.monash.edu (A.M.M.); nhi.tran2@student.rmit.edu (N.T.T.); deborah.deguingand@hudson.org.au (D.L.d.G.); robert.Galinsky@hudson.org.au (R.G.); 2 Department of Obstetrics and Gynaecology, Monash University, Clayton, VIC 3800, Australia; kirsten.Palmer@monash.edu; 3 School of Health & Biomedical Sciences, RMIT University, Bundoora, VIC 3082, Australia; david.walker@rmit.edu.au; 4 Institute for Physical Activity and Nutrition, School of Exercise and Nutrition Sciences, Deakin University, Geelong, VIC 3220, Australia; mphilipgabriel@deakin.edu.au (M.P.); paul.dellagatta@deakin.edu.au (P.A.D.G.); rod.snow@deakin.edu.au (R.J.S.); 5 Division of Neuroscience, Oregon National Primate Research Center, Beaverton, OR 97006, USA; shermanl@ohsu.edu; 6 Department of Cell, Developmental and Cancer Biology, Oregon Health & Science University, Portland, OR 97239, USA; 7 Division of Reproductive & Developmental Sciences, Oregon National Primate Research Center, Beaverton, OR 37009, USA; kellehem@ohsu.edu; 8 Capital and Coast District Health Board, Department of Paediatrics, University of Otago Wellington, Wellington 6242, New Zealand; max.berry@otago.ac.nz

**Keywords:** creatine, nutritional supplements, fertility, pregnancy, newborn, development, brain injury

## Abstract

Creatine metabolism is an important component of cellular energy homeostasis. Via the creatine kinase circuit, creatine derived from our diet or synthesized endogenously provides spatial and temporal maintenance of intracellular adenosine triphosphate (ATP) production; this is particularly important for cells with high or fluctuating energy demands. The use of this circuit by tissues within the female reproductive system, as well as the placenta and the developing fetus during pregnancy is apparent throughout the literature, with some studies linking perturbations in creatine metabolism to reduced fertility and poor pregnancy outcomes. Maternal dietary creatine supplementation during pregnancy as a safeguard against hypoxia-induced perinatal injury, particularly that of the brain, has also been widely studied in pre-clinical in vitro and small animal models. However, there is still no consensus on whether creatine is essential for successful reproduction. This review consolidates the available literature on creatine metabolism in female reproduction, pregnancy and the early neonatal period. Creatine metabolism is discussed in relation to cellular bioenergetics and de novo synthesis, as well as the potential to use dietary creatine in a reproductive setting. We highlight the apparent knowledge gaps and the research “road forward” to understand, and then utilize, creatine to improve reproductive health and perinatal outcomes.

## 1. Introduction

Female reproductive organs are some of the most regenerative and highly energetic tissues within the body [1]. As such, there is an undeniable link between energy metabolism and reproductive success. By understanding the intricacies of energy metabolism and adenosine triphosphate (ATP) production throughout female reproduction, we are best placed to address irregularities that may contribute to infertility and poor pregnancy outcomes.

**Figure 1:**
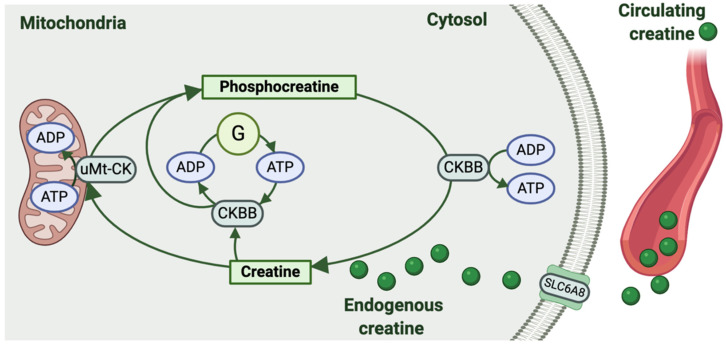
The creatine kinase circuit. Creatine can be produced endogenously by some cells or taken up from the circulation via the creatine transporter (*SLC6A8*). Creatine is then phosphorylated from adenosine triphosphate (ATP) to form phosphocreatine and adenosine diphosphate (ADP) in a reaction catalyzed by ubiquitous mitochondrial creatine kinase (uMt-CK). Isoforms of creatine kinase in the cell cytosol (mainly brain-type creatine kinase CKBB) are also linked to glycolytic enzymes (G) to generate phosphocreatine and ADP from glycolytic ATP. Then, when required for energy-dependent cellular processes, cytosolic isoforms of creatine kinase (CKBB) hydrolyze the bond between creatine and the phosphate group stored as phosphocreatine, thus regenerating ATP and creatine. Note: muscle-type cytosolic creatine kinase (CKMM) expression has also been detected in mouse oocytes.

To sustain high energy levels, cells, including those of our reproductive tissues, are equipped with high energy phosphagens [2]. While invertebrates use a variety of different phosphagen systems, the creatine kinase circuit is the sole phosphagen system of higher vertebrates [3]. Ultimately, the creatine kinase circuit serves as an immediate temporal energy buffer, maintaining ATP turnover and the intracellular ATP/adenosine diphosphate (ADP) ratio. It also provides spatial energy buffering by providing for the transport of high-energy phosphates from sites of ATP production (i.e., oxidative phosphorylation and glycolysis) to sites of ATP utilization within the cytosol (Figure 1) [4].

**Figure 2:**
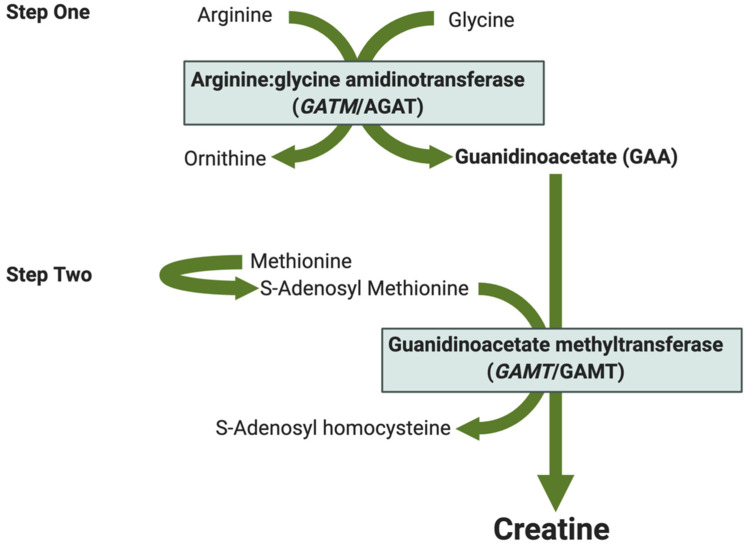
Schematic of de novo creatine synthesis. Step one—Arginine and glycine combine to form guanidinoacetic acid (GAA) and ornithine in a reaction catalyzed by arginine:glycine amidinotransferase (AGAT translated from the *GATM* gene). Step two—GAA is methylated to form creatine and S-Adenosyl homocysteine. S-adenosyl methionine (SAM) is the primary methyl donor for this reaction catalyzed by guanidinoacetate methyltransferase (*GAMT*/GAMT).

Creatine is the substrate for the creatine kinase circuit. It can be endogenously synthesized by the body or acquired from a diet containing meat, fish, dairy products or over-the-counter nutritional supplements [5]. De novo creatine synthesis involves a two-step process (Figure 2). First, the enzyme L-Arginine: glycine amidinotransferase (AGAT, translated from the *GATM* gene) catalyzes the production of guanidinoacetate (GAA), the creatine precursor, and ornithine from arginine and glycine. In the second step, guanidinoacetate N-methyltransferase (GAMT) catalyzes the methylation of GAA producing creatine and S-Adenosyl homocysteine.

Circulating creatine is taken up by cells via a sodium-dependent creatine transporter encoded by the *SLC6A8* gene (Figure 1) [6,7]. Reversible phosphorylation of intracellular creatine by the ubiquitous mitochondrial creatine kinase (uMt-CK) or cytosolic creatine kinases linked to glycolytic enzymes produces the high energy compound, phosphocreatine [8]. Promoted by shifts in the intracellular ADP/ATP ratio, cytosolic isoforms of creatine kinase then hydrolyze the bond between creatine and the stored phosphate group, regenerating ATP (Figure 1) [4]. There are two cytosolic isoforms of creatine kinase that have been identified in female reproductive tissues. The most prominent is the brain-type creatine kinase (CKBB), with the muscle-type creatine kinase (CKMM) isoform also being identified in the mouse oocyte [9]. Overall, the creatine kinase circuit produces ATP more rapidly than any other metabolic system, with the interplay between the different components of creatine metabolism being essential to sustain the bioenergetic demands of a cell [3].

Our understanding of creatine metabolism in reproduction and pregnancy is growing. Indeed, review of the literature sees creatine metabolism increasingly appear throughout studies of both male and female reproduction. This association extends from observational studies published in the early 20th century through to more recent non-targeted metabolomic screens [10,11]. There is now strong evidence that alterations to creatine homeostasis occur with the normal progression of a healthy female reproductive cycle and with pregnancy [11,12]. Altered creatine metabolism has also been linked to reduced fertility and specific pregnancy-related pathologies [13,14]; however, there is no clear consensus as to whether creatine metabolism is indeed an essential component of bioenergetics for successful reproduction.

This review aims to consolidate the available literature, old and new, pertaining to creatine metabolism in reproduction, pregnancy, fetal brain development, and the early neonatal period. Creatine metabolism will be discussed in relation to its capacity to maintain cellular bioenergetics, the ability of reproductive tissues to synthesize creatine de novo, and the potential to use dietary creatine as a protective treatment against the effects of in utero fetal hypoxia and perturbations in newborn brain metabolism. We will highlight the apparent knowledge gaps and the research “road forward” to ultimately understand, and then potentially harness, creatine metabolism to improve reproductive health and perinatal outcomes.

## 2. Creatine Metabolism in the Female Reproductive System

### 2.1. Oocytes and Surrounding Cells

The oocyte requires large amounts of energy during its development in preparation for fertilization. There are also energy reserves stored within the mature oocyte to facilitate the initial period of embryogenesis [15]. Creatine metabolism occurs within human and mouse oocytes. These cells contain phosphocreatine (~4 to 5 mmol.kg^−1^ dry mass) with an equal level of creatine [16]. They also express creatine kinase (CK) genes and proteins, displaying high levels of in vitro CK activity [16,17]. The available data on CK expression and activity suggest that the use of the creatine kinase circuit to generate ATP by the oocyte may be species-specific. In mice, cytosolic creatine kinase (CKBB) activity increased 5-fold during oocyte maturation. This same study found that CKBB activity increased after fertilization, up to the stage of an eight-cell embryo, before a steep decline as the embryo reached the blastocyst phase [17]. These findings were confirmed by a further study of mouse embryos completed by Forsey et al. (2013) [18]. A more recent study, also in mice, found that mature oocytes have increased cytosolic creatine kinase (*CKMM*) gene expression when exposed to human chorionic gonadotropin (hCG) stimulation; the hormone produced by trophoblast cells after fertilization [9]. However, these same cells lacked expression of the *uMt-CK* gene, bringing into question the functional capacity of the creatine kinase circuit to produce phosphocreatine and then ATP, within the experimental paradigm used [9]. Further studies focused on protein expression and CK enzyme activity are still required. In contrast, both uMt-CK and cytosolic (CKBB) gene and protein expression have been detected in bovine oocytes. Scantland et al. (2014) observed that, compared to mature bovine oocytes, immature oocytes had higher gene expression of the creatine kinases, and that when oocytes were matured in a medium containing specific CK inhibitors, they displayed an elevated intra-oocyte ADP:ATP ratio [19]. These findings indicate that CK activity is present in oocytes and that the creatine kinase circuit is used to help maintain intracellular ATP levels.

The source of creatine for the oocyte (endogenous synthesis or cellular up-take) remains unclear. A study by Fezai et al. (2015) found that oocytes from Xenopus laevis (African clawed frog) do not transport creatine across their plasma membrane, concluding that these cells do not express creatine transporter proteins [20]. However, caution should be taken when extrapolating amphibian to mammalian physiology as high levels of creatine transporter gene (*SLC6A8*) expression have been reported in the ovaries of rats, suggesting that at least some ovarian cells may contain the transporter [21]. It is also important to note that for some metabolites, passive diffusion into the oocyte from attached surrounding support cells (e.g., cumulus cells) has been documented and one cannot rule out the possibility that the same mechanism supplies the oocyte with creatine.

Indeed, creatine metabolism has been reported in the specialized somatic cells that surround the mammalian oocyte. Collectively, these cells support oocyte maturation, facilitate fertilization and subsequent development into a viable embryo. Human ovarian stromal cells, in particular, may contribute to de novo creatine synthesis via GAA production, with these cells having detectable levels of the *GATM* gene and AGAT protein [22]. However, *GAMT* gene but not protein expression was detected in these same cells, indicating that they are unable to methylate GAA to produce creatine [22]. The enzymes of the creatine kinase circuit are present within cumulus cells signifying the presence of creatine metabolism in these specialized granulosa cells which lie directly adjacent to the oocyte. The activity of the CK enzymes appears to be low and remain unchanged with hormone stimulation, so there is no current evidence for creatine metabolism varying across the ovarian cycle in these specific cells [9]. Interestingly, *CKBB* gene expression appears elevated in cumulus cells acquired from women undergoing assisted fertility treatment who were either older than 38 years of age or younger than 28 years of age, with the level of expression positively associated with good-quality embryos in both younger and older women [23]. Finally, research investigating the metabolites secreted from bovine cumulus–oocyte complexes (COCs) during in vitro maturation showed a substantial increase in creatine (~ 450-fold) and a comparatively modest increase (~ 2-fold) in GAA concentration in the maturation medium bathing the cells. Moreover, both creatine and GAA are detectable within bovine cumulus cells [24]. Further experiments demonstrated that the addition of creatine alone to the maturation medium did not affect the developmental competence of the oocyte [24]. The secretion of creatine from the COCs is therefore likely performing another role such as facilitating sperm function. Indeed, there is considerable research on the importance of creatine metabolism for optimal sperm motility, hyperactivation and capacitation in preparation for fertilization [25].

### 2.2. Follicular Fluid, the Oviduct and Oviductal Fluid

Follicular fluid is derived from plasma and secretions synthesized in the follicle wall [26]. This fluid contains metabolites, which are critical for oocyte growth and development [27]. Creatine is present in human follicular fluid [28,29]. Interestingly, the creatine levels in follicular fluid are significantly lower in women with ovarian endometrioma compared with controls [30]. Umehara et al. (2018) demonstrated that mouse follicular fluid creatine concentrations increased markedly around ovulation [9]. This is in contrast to equine follicular fluid, where creatine levels were unchanged with follicular development or near ovulation [31]. The data from Umehara et al. (2018) also suggest that the increase in follicular fluid creatine levels likely resulted from an increase in creatine synthesizing capacity within the granulosa cells of the ovary because they displayed a significant increase in *GATM* and *GAMT* gene expression with equine chorionic gonadotropin stimulation around the time of ovulation [9]. Further studies are needed to track human follicular fluid creatine levels across the ovarian cycle to better understand the importance of creatine metabolism for oocyte growth and development.

Fertilization, the process of the mature oocyte and sperm fusing to give rise to the embryo, occurs within the oviduct. This structure is lined with an epithelium coated by an oviductal fluid, composed in part by secretions from these cells and in part by blood plasma filtrate. The oviductal fluid composition is species-specific, but overall contains proteins, hormones, growth factors and metabolites that vary depending on the stage of the reproductive cycle and also on the presence of gametes or embryos. To the best of our knowledge, creatine, phosphocreatine, and GAA levels have not been determined in human oviductal fluid. Gene expression of the creatine synthesizing enzymes *GATM* and *GAMT* has been measured in the human and rat oviduct [21,22], but neither protein was detected in a study using immunohistochemical analysis [22]. In partial agreement, GAMT gene and protein were not found to be expressed in the mouse oviduct [32]. There are also no human data on the protein expression levels of the creatine transporter in the oviduct tissue; however, the creatine transporter gene (*SLC6A8*) is expressed in both human and rat oviducts [21,22]. Further analysis is obviously required to adequately characterize creatine metabolism in the human oviduct, and its role in the bioenergetics of fertilization. Studies in other mammalian species provide some further information on creatine metabolism in the oviduct and oviductal fluid. For example, the creatine concentration in equine oviductal fluid is very high (3–4 mM) compared with plasma levels (8–103 µM) and does not change pre- to post-ovulation [33,34]. Creatine levels in mouse oviductal fluid increased with hCG stimulation although this was not associated with an increase in *GATM* or *GAMT* gene expression in oviduct cells [9]. Consequently, the source of the elevated creatine levels (endogenous synthesis or cellular up-take) found in the oviductal fluid is currently unclear. Interestingly, mice sperm cultured in in vitro fertilization (IVF) medium supplemented with creatine displayed elevated ATP levels and increased motility [9]. A similar observation has recently been reported in pig IVF studies [35]. These findings raise the possibility again that increased creatine levels in the female reproductive tract are taken up by sperm, contributing to their hyperactivation and increasing the chance of successful fertilization [25]. Whether the same is true for human sperm and whether the simple addition of creatine to IVF medium can improve outcomes for couples undertaking artificial reproductive therapies warrant further investigation.

### 2.3. The Endometrium

There is evidence that the creatine kinase circuit is active in uterine tissue [36,37], and that components of this metabolic system change throughout the female reproductive cycle and with pregnancy [38]. The use and regulation of this system in the uterus are not well understood; however, it is likely to be significant given that up-regulation of creatine metabolism in uterine tissues appears to correlate with phases of increased uterine energy demand throughout the female reproductive cycle, pregnancy, and parturition.

Research explicitly examining human endometrial tissue reports that components of creatine metabolism are up-regulated during the secretory phase of the menstrual cycle [39]. During this phase when embryo implantation can occur, endometrial tissue contains creatine and displays increased expression of the creatine transporter gene (*SLC6A8*), as well as an up-regulation of cytosolic creatine kinase (*CKBB*) gene expression and enzyme activity [37,39,40,41]. This increase in CKBB enzyme activity occurs in both stromal cells and endometrial glands, but is much higher in the latter [37]. Recently, the increased CKBB protein expression has been localized to the apical surface of the human endometrial glandular and luminal epithelial cells [42]. This raises the possibility that CKBB activity, and therefore production of ATP from phosphocreatine stores, may be necessary for regulating energy homeostasis during the receptive phase of the menstrual cycle [11]. Whether endometrial creatine synthesis also changes across the cycle has not been investigated in humans. However, some aspects of creatine synthesis in the endometrium have been linked to reduced fertility in rodents, with AGAT knockout female mice proving to be infertile [43]. It is unclear if infertility is directly linked to endometrial function or another component of the reproductive cycle. It is also unclear if untreated AGAT deficiency leads to infertility or poor pregnancy outcomes, but this warrants further investigation [44].

Components of endometrial creatine metabolism are also altered during pregnancy [36,45,46,47,48]. In the pregnant rodent, uMt-CK and CKBB protein are highly expressed in the decidua parietalis and basalis, with these enzymes needed to complete the creatine kinase circuit mainly located within stromal cells close to the multiple sites of placental implantation [36]. Surprisingly, very little is known about creatine kinase gene and protein expression in the human endometrium during pregnancy. Only one study has attempted to investigate this, reporting that creatine kinase activity was present in human decidual explants obtained at term [46]. In regard to creatine synthesizing capacity, one rodent study [48] has reported that AGAT activity was high in the uterine decidua during pregnancy. However, there was little or no GAMT enzyme activity present in the endometrium of these animals. These findings suggest that the decidua in pregnant rodents has a high capacity to produce GAA but does not complete the methylation step to produce creatine. A similar finding of increased uterine GAA production has also been noted in pregnant sheep [49]. It is currently unknown what adaptations in creatine synthesis capacity, if any, occur in the human endometrium with pregnancy. Furthermore, characterization of endometrial creatine, phosphocreatine or GAA levels during human pregnancy remains to be established.

### 2.4. The Myometrium

The human non-pregnant myometrium displays creatine kinase activity [50] and phosphocreatine at a low level compared to human pregnant myometrium [51]. Cultured human uterine smooth muscle cells are capable of importing extracellular creatine using a mediated process, suggesting that creatine transporter proteins are present in these cells [52]. However, there have been no studies exploring whether the human myometrium in the non-pregnant state can produce GAA or creatine. It is also not known if the myometrial expression of creatine kinase isoforms is altered during the female reproductive cycle.

There is evidence that creatine metabolism is up-regulated in the myometrium during pregnancy [51,53,54,55,56]. Phosphocreatine levels are increased in the human pregnant myometrium at term compared to non-pregnant tissue [55]. This likely acts as an increased energy reserve for the uterus during labor [57]. The mechanism(s) leading to the increased phosphocreatine levels in the myometrium during pregnancy remain unclear but are likely due to a concomitant increase in the total creatine content. Currently, no evidence demonstrates the presence of the creatine transporter, or synthesizing enzymes AGAT and GAMT in the human pregnant myometrium. Consequently, it is not known if the myometrium is capable of transporting creatine into cells, or whether myometrial cells can produce GAA and/or creatine during pregnancy. Cytosolic creatine kinase (*CKBB*) gene expression has been measured in human pregnant myometrium and at term is three-fold higher than earlier in gestation [53]. However, the underlying mechanism for this increase is unknown. Additionally, there are no existing data for CKBB protein, nor uMt-CK gene and protein expression in human myometrium during pregnancy, so the functional consequences of these gene expression changes and the overall use of creatine to sustain myometrial ATP production remain unclear. This should be an area of focus for future research, as it is highly plausible that creatine metabolism in the myometrium is important for optimal contractile performance during labor [38]. Studies on creatine metabolism in the female reproductive tract are summarized in Table 1.

## 3. Creatine Metabolism in the Human Placenta

Optimal placental function is required to ensure both the successful maintenance of pregnancy, as well as fetal growth and development [58,59]. As such, the human placenta is a highly metabolic organ, consuming 40–60% of oxygen and glucose transported to the uterine cavity [60]. This energy consumption serves two purposes: [1] growth of the placenta itself (placental tissue turnover is 3–4 g a day, or 1–2% of its total mass); [2] nutrient transfer, waste transport, and peptide and steroid hormone production for fetal growth and development [61]. Consequently, pregnancy encompasses large changes in maternal glucose, carbohydrate, amino acid, lipid, and fatty acid-derived energy metabolism to meet placental and fetal requirements [62,63]. There is growing evidence that creatine metabolism should be added to the list of pathways needed to maintain cellular bioenergetics in both the healthy and metabolically compromised placenta.

The human placenta expresses the mitochondrial (uMt-CK) and cytosolic (CKBB) isoforms of creatine kinase, with expression patterns varying throughout the three trimesters of pregnancy. At a gene level, *uMt-CK* and *CKBB* mRNA expression appear low in the first and second trimester before a large peak closer to term [64]. In this study by Thomure et al. (1996), post-transcriptional regulation of both creatine kinases was apparent with CKBB protein expression remaining consistent throughout gestation and uMt-CK protein rising through to mid-gestation before declining just before term. Overall, this biphasic expression correlates with the metabolic activity of the placenta and suggests that the creatine kinase circuit contributes to placental metabolism during pregnancy [64].

It has also been reported that the human placenta has the enzymatic machinery to synthesize GAA and creatine [65]. The capacity for placental creatine synthesis and transport is likely in place from early gestation, with first-trimester chorionic villous biopsies (10–13 weeks’ gestation) expressing *GATM*, *GAMT* and *SLC6A8* mRNA [13]. Assessment of placental tissue collected at term confirmed that the human placenta expresses AGAT, GAMT and SLC6A8 at both the gene and protein level. The AGAT protein is localized to the stromal and endothelial cells of the fetal capillaries, whereas GAMT is predominantly located on the apical side of multinucleated syncytiotrophoblast cells [65]. The creatine transporter is also located on these highly specialized cells at the maternal–fetal interface, which are the site of glucose, amino acid, and fatty acid transfer from maternal blood into the fetal circulation [62,65,66]. This continuous epithelial barrier in the outer layer of all placental villi has a high metabolic demand, particularly during late gestation [67]. Whether the location of the creatine transporter and enzyme responsible for the methylation of GAA to creatine facilitates maternal/fetal transfer of creatine or supports the metabolic activities intrinsic to syncytiotrophoblast function, or both, is yet to be fully determined.

Inadequate placental perfusion and subsequent metabolic compromise are hallmarks of several common obstetric complications, including preeclampsia (PE), gestational diabetes, and fetal growth restriction (FGR). Thus, in addition to the adaptations of increasing energy demands during a healthy pregnancy, the placenta often must respond to homeostatic challenges, such as acute and chronic hypoxic insults, throughout gestation [68]. Investigations of creatine metabolism in metabolically unstable placentae revealed that there might be an increased reliance on the creatine kinase circuit to maintain ATP homeostasis under sub-optimal conditions. Indeed, increased levels of phosphocreatine have been detected in placentae from pregnancies occurring at high altitude, where the oxygen in air is chronically reduced from 21% to ~18% [69]. In a study of FGR placentae, total creatine content was increased by 43%, and creatine transporter (*SLC6A8*) mRNA expression was increased by two-fold in the third trimester compared to gestation-matched healthy controls [13]. These changes occurred despite no differences in creatine synthesizing enzyme (AGAT and GAMT) protein expression, and there were no differences in creatine concentrations in either maternal or venous cord serum at delivery [13]. It should also be noted that expression patterns of the creatine transporter, creatine synthesizing enzymes and creatine kinases did not differ in early gestation between pregnancies that delivered appropriately grown babies in comparison to small for gestational age. As such, the authors postulate that it is the progressive nature of placental insufficiency, and changes in intracellular creatine content or ADP/ATP ratios that likely steer the changes in creatine metabolism observed in the third-trimester placenta [13]. This timing coincides with the peak metabolic rate of the placenta, when placental insufficiency may become most detrimental to the developing fetus and places the fetus at further disadvantage in terms of its ability to tolerate the physiological stress of labor [70].

Similarly, in PE placentae, total creatine content has been reported to increase by 38%, with *GATM*, *GAMT*, *SLC6A8* and *CKBB* mRNA expression also significantly increased compared to gestational age-matched controls, although, again, these differences were not observed at a protein level [14]. There is evidence that, in the case of PE, this additional creatine may be transported to the compromised fetus, with a recent study by Jääskeläinen et al. (2018) reporting an increase in creatine concentration in venous cord plasma from PE pregnancies [71]. Some interesting correlations were also observed in healthy control placentae throughout these collective retrospective studies, with placental *GATM* mRNA expression and GAA tissue content decreasing with advancing gestational age and birth weight. These adaptations associated with placental senescence were not observed in FGR or PE placentae, indicating an ongoing reliance on the creatine kinase circuit for placental bioenergetics in compromised pregnancies [13,14]. It is interesting to note that *GATM*, the gene that expresses AGAT, has been identified as a maternally imprinted gene, and thus is exclusively expressed in placental tissue from the maternal allele [72]. Imprinted genes are often associated with regulation of energy exchange between the mother and developing embryo, and are thought to restrain the over-allocation of maternal resources to the fetus [73]. It is postulated that high levels of AGAT expression in the placenta, and thus potential for creatine synthesis through the production of GAA, may protect the mother from dynamic shifts in the energy required to sustain embryonic or fetal development [73]. Indeed, a study by McMinn et al. (2006) investigating changes to the expression of maternally imprinted genes in the term human placenta identified a down-regulation in *GATM* mRNA in samples from FGR pregnancies (see Table 2) [74].

With consideration of the metabolic demands of the human placenta, it is not hard to rationalize the use of the creatine kinase circuit to support placental bioenergetics. Together, studies to date indicate that the hypoxic placenta may have an increased reliance on creatine and the creatine kinase circuit to buffer spatial fluctuations in ATP homeostasis. This increased capacity to re-phosphorylate ADP via an oxygen-independent pathway may help maintain the high metabolic rate of the third-trimester placenta, and the myriad of ATP-dependent processes required to sustain pregnancy, including synthesis of structural proteins, enzymes, and a wide range of products with important endocrine, hemostatic and immunological functions [75,76]. However, what remains to be understood is the trigger for these adaptations, as well as the consequences they have on both the placenta and the fetus. Defining the mechanisms associated with placental hypoxia in terms of metabolic changes may explain what drives adaptations in placental creatine metabolism. Importantly, placental mitochondrial changes are characteristic of common pregnancy stress conditions such as maternal diabetes, obesity, PE, and hypoxia [77,78,79,80]. Hence, the metabolic adaptation to increase intracellular placental creatine stores in the context of mitochondrial function warrants further investigation.

## 4. Maternal Creatine Metabolism during Pregnancy

As pregnancy progresses and the fetus grows, maternal metabolism shifts with several adaptations required to meet the changing metabolic demand of advancing gestation [64]. As detailed in this review, the role of creatine metabolism in contributing to cellular bioenergetics during reproduction and pregnancy is becoming evident. With provisions of creatine being required to accommodate rapidly expanding tissue beds within the uterus, placenta and fetus, one must also consider the source of the additional creatine required throughout gestation to optimally meet these demands. Data from both human and animal studies have established that pregnancy modifies maternal creatine homeostasis, that maternal characteristics are associated with circulating creatine concentrations during gestation, and that alterations to maternal creatine homeostasis throughout gestation may be linked to the growth and well-being of the offspring.

Adaptations to maternal creatine metabolism throughout pregnancy were first characterized extensively in a study of pregnant spiny mice. This rodent undergoes in utero organ maturation on a similar trajectory to humans [81]. This study mapped changes in maternal circulating creatine, synthesis, excretion, transport, and storage across gestation [12]. They found that maternal plasma creatine concentrations fell progressively from mid to late gestation, with levels in pregnant spiny mice being significantly lower than in non-pregnant controls at all time points analyzed. Urinary excretion of creatine also decreased from mid to late gestation and was significantly lower compared to non-pregnant female spiny mice. Pregnancy was associated with increased *GATM* mRNA and AGAT protein expression in the maternal kidney, considered a primary site of GAA production. This may indicate an up-regulation of creatine synthesis with pregnancy, as renal AGAT activity is also considered a rate-limiting step of creatine production [12]. Increased creatine transporter (*SLC6A8*) mRNA expression was observed in maternal tissues with high-energy demand, such as the heart and skeletal muscle at term. In contrast, creatine transporter expression was decreased in the maternal brain and liver. These changes suggest this may be an adaptive mechanism that ensures creatine is available to maternal tissues where energy expenditure can be high. Indeed, the creatine content of the maternal heart and kidney was increased at term, compared to levels observed in non-pregnant tissues [12]. Overall, these results indicate changes to maternal creatine homeostasis in the spiny mouse may be a fundamental physiological adaptation to pregnancy.

Changes in maternal plasma and urinary creatine have also been reported in human pregnancy. The normative range of plasma creatine is reported to be 35.6 μM ± 15.15 during pregnancy, which is ~35% lower than the normal range of 54.8 μM ± 21.0 for non-pregnant females [82,83]. Conversely, urinary creatine excretion rises from 46 (9–135) μmol/mmol creatinine in a non-pregnant state [84] up to 146.7 (58–273) μmol/mmol creatinine during pregnancy, a 3-fold increase (Ellery et al., unpublished data). Unlike the spiny mouse, multiple human studies have reported that maternal plasma creatine levels, while low, remain stable throughout gestation but consistently between species, the rate of urinary creatine excretion declines with advancing gestation [85,86]. Collectively, these data suggest that there is an increased requirement for maternal creatine due to the rapid growth and increased metabolic requirements of the fetus in the third trimester of pregnancy.

In a retrospective study, associations were identified between key maternal characteristics and circulating and excreted creatine levels during pregnancy [85]. Maternal smoking was positively associated with plasma creatine levels, whereas parity (having previously given birth) had a negative association. The study also found that maternal body mass index (BMI) and asthma were positively associated with urinary creatine, whereas maternal urinary creatine excretion across pregnancy was positively correlated with birth weight centile and birth length, suggesting a relationship between maternal creatine status and fetal growth [85]. This notion is supported by a study dating back to 1913, where increases in newborn body weight were shown to be roughly proportional to the creatine excreted in the urine by the mother at term [10]. Thus, the regulation of creatine acquisition, its loss via urinary excretion, and its delivery to the fetus across the placenta may be important determinants of fetal growth and development. Whether alterations in maternal circulating creatine concentrations are indicative of other poor perinatal outcomes is still to be ascertained. In a retrospective case-controlled study, an 18% reduction in maternal serum creatine concentration during the third trimester of pregnancy was associated with a greater incidence of poor perinatal outcomes, which was defined by a composite measure of small for gestational age, preterm birth and admission to neonatal intensive care [87]. A recent case study of a pregnant female with an AGAT deficiency (inability to synthesize creatine) reported that the patient required adjustment of her dietary creatine treatment during pregnancy when sonographic monitoring at mid gestation indicated a reduction in fetal growth associated with a decline in the patient’s plasma and urinary excreted creatine concentrations. After increasing the patient’s dietary creatine supplement from 2 g to 3 g daily, she delivered a healthy infant at 35 weeks’ gestation on the 25th centile for birth weight, with normal brain creatine levels. The infant achieved typical developmental milestones at one year of age, when the case study ceased [44].

Thus, while clear links are beginning to emerge between maternal creatine homeostasis throughout pregnancy and infant outcomes, further studies are required to better understand adaptations to maternal creatine homeostasis throughout gestation and their association with pregnancy outcomes. In particular, due to the importance of dietary creatine to maintain circulating creatine levels and augmenting endogenous synthetic capability, understanding habitual dietary preferences and basal nutritional status of women is essential to optimize pregnancy wellbeing. A prospective longitudinal cohort study (the Creatine and Pregnancy Outcomes (CPO) study) has been undertaken to address these knowledge gaps [88].

## 5. Fetal Creatine Metabolism and Use of Supplementary Creatine to Prevent Perinatal Brain Injury

The creatine kinase circuit is thought to play an essential role in energy homeostasis during embryonic development, particularly the development of the central nervous system (CNS) [89,90]. This idea was first explored by Braissant et al. (2005), in a study which described embryonic gene and protein expression of AGAT, GAMT and the creatine transporter (SLC6A8) in numerous tissue types in the rat from early in gestation [91]. From a neurodevelopmental perspective, creatine metabolism has been linked to the growth of dendrites and axons, and migration of neural growth cones [92,93].

The importance of creatine metabolism for cellular bioenergetics in the fetal brain is further supported by a recent in utero fetal magnetic resonance spectroscopy (^1^H-MRS) study that clearly illustrated cerebral creatine accretion with advancing gestation [94]. This study by Evangelou et al. (2015) examined 204 spectra obtained from fetuses of 129 pregnant women and found fetal cerebral creatine concentrations more than doubled between 18 and 40 weeks’ gestation [94]. Indeed, the brain possesses both uMt-CK and CKBB creatine kinases, and a significant amount of cerebral ATP is generated via the creatine kinase circuit [95]. CK isoforms have been found within specific cells of the hippocampus (granular and pyramidal cells), the cerebellum and choroid plexus [96]. This suggests a need for these cell types to use creatine to maintain ATP turnover. Additionally, transfer of creatine between cells is made possible by the expression of the creatine transporter on neurons, oligodendrocytes and astrocytes [92,97]. The entry of circulating creatine into the adult brain appears to be limited to some extent by the blood–brain barrier [98]; however, the creatine synthesizing enzymes, AGAT and GAMT are expressed in varying levels by developing and mature neurons, astrocytes and oligodendrocytes [99], as well as low levels by microglia [100], meaning the brain is able to synthesize creatine endogenously. Indeed, it has been argued that the brain is not reliant on exogenous or systemic endogenous sources of creatine at all, and that cells within the brain can synthesize adequate amounts of creatine to maintain function [101], but whether this holds true for the immature and rapidly developing perinatal brain is yet to be ascertained.

Similar to the axons of neurons in the developing brain, oligodendrocytes, which supply myelin sheaths to axons in the CNS, and oligodendrocytes progenitor cells have high energy demands and are highly susceptible to perinatal energy deprivation (e.g., [102]). Indeed, perinatal brain injury can lead to significant neurological impairments as a result of dysmyelination or delayed myelination linked to the loss of oligodendrocyte progenitor cells that fail to mature into myelinating oligodendrocytes [103]. Although AGAT is expressed by neurons, astrocytes, and oligodendrocytes, GAMT is primarily expressed by oligodendrocytes [92,104], suggesting that oligodendrocytes are a major source of endogenous creatine in the CNS. Indeed, mutations in the creatine synthesizing genes, *GATM* and *GAMT*, demonstrate that impaired myelination [105] and *GAMT*-deficient mice have impaired remyelination and oligodendrocyte apoptosis following a demyelinating insult [104]. These studies highlight the importance of creatine in myelination and remyelination and indicate that oligodendrocytes are likely a major target of supplemental creatine. An initial study completed in pregnant rats found that supplementing the maternal diet with 1% creatine for 10 days prior to delivery improved morphological and electrophysiological development of cornu ammonis (CA1) neurons in the offspring within the first three weeks of life [106].

In addition to creatine metabolism supporting the brains’ basal metabolic function, growth and maturation, creatine can maintain ATP turnover, acid-base balance and mitochondrial function. This, together with its antioxidant, vasodilator, and anti-excitotoxic properties, makes it a candidate for the treatment of ischemic–reperfusion brain injuries [107]. In the adult setting, studies have focused on neurodegenerative diseases including Alzheimer’s, Parkinson’s and Huntington’s disease, as well as Amyotrophic lateral sclerosis [108]. Much of this work to date has also focused on fetal and neonatal brain injury [109,110,111,112]. For example, in brain slices prepared from neonatal mice and fetal guinea pigs, the ex vivo addition of creatine preserved ATP turnover and reduced neuronal injury [113,114]. Early in vivo studies in rats also demonstrated that a low phosphocreatine/creatine ratio correlated with a higher susceptibility of the immature rat to experience hypoxic seizures early in development [115]. This same study reported that creatine supplementation improved survival and prevented seizure activity [115]. Similar findings were made in a study of rabbit pups [116]. Finally, a study in immature rat pups found that subcutaneous injections of creatine administered in the neonatal period prevented brain edema associated with severe hypoxia–ischemia [117].

Following on from these studies, the neuroprotective role of creatine was investigated in the context of intrapartum (birth) asphyxia using the precocial spiny mouse. In this model, pregnant spiny mice were fed a creatine supplemented diet (5% *w*/*v*) from mid-gestation (day 20 of gestation), which resulted in creatine loading of the fetal brain, heart, liver and kidney at term (day 39 of gestation) [115,118]. In the offspring of control fed dams, intrauterine hypoxia of 7.5–8 min at term was associated with an increase in pro-apoptotic protein BAX, cytoplasmic cytochrome *c*, and caspase-3 in the fetal brain and high perinatal mortality rates [115]. Conversely, Ireland et al. (2008 and 2011) reported that maternal creatine supplementation mitigated neurological injury in the fetal brain and was associated with increased pup survival and improved postnatal growth [115,118]. In addition to specifically protecting the brain, these studies of intrapartum asphyxia in the spiny mouse model also reported that maternal dietary creatine supplementation during gestation had beneficial effects for other organs involved in the multi-organ pathology of intrapartum asphyxia. For example, maternal creatine supplementation prevented structural and functional damage to the diaphragm [119,120] and skeletal muscle [121], and prevented acute kidney injury in the neonatal period [122], as well as the risk of developing chronic kidney disease later in life (Table 3) [123]. These findings are significant as it has been reported that for 70% of cases of newborn brain injury following intrapartum hypoxia, the primary and direct cause of hypoxic–ischemic encephalopathy (HIE) with neurological injury may not be cerebral oxygen deprivation per se, but rather, injury developing secondary to multi-organ injury [124]. The capacity of maternal dietary creatine supplementation to protect against multiple organ injury following intrapartum hypoxia makes it a unique candidate treatment within the perinatal asphyxia landscape and one worth exploring further. Limitations to the in vivo and rodent studies completed to date include the restricted ability to assess the pathophysiological response to in utero hypoxia with creatine supplementation and the effects of hypoxia and creatine loading in discrete brain regions. Pre-clinical studies in translational large animal models are currently underway to overcome these limitations and fully ascertain the capacity of maternal dietary supplementation during pregnancy as a preventative strategy for hypoxia-induced perinatal brain injury [125].

## 6. Creatine Metabolism in the Neonate, with a Focus on the Potential Consequences of Preterm Birth

Creatine and an effective creatine kinase circuit is critical for brain metabolism [126]. Postnatal studies have identified that an increase in cerebral creatine content occurs during the first three months of life amongst infants born at term [127,128]. The importance of this cerebral creatine accretion for optimal brain development is made evident by those infants diagnosed with inherited creatine deficiency syndromes (CDS). During fetal life, and in the immediate newborn period, these infants are symptom-free as creatine requirements have been met through maternal/placental supply. Following birth, they become progressively creatine deficient [129] with progressive manifestation of neurological symptoms, including impaired psychomotor function and seizures [130]. Importantly, dietary creatine supplementation is proving to be a relatively simple solution to certain forms of CDS, specifically AGAT- and GAMT-deficiency disorders, enabling restoration of cerebral creatine, thereby allowing young children with these conditions to thrive. However, much still needs to be done to improve awareness of CDS in the wider community and promote early screening of at-risk infants [131].

As creatine and phosphocreatine are spontaneously broken down into creatinine at a rate of 1.7%/day, we all have a requirement to replenish our creatine and phosphocreatine stores either through our diet or endogenous synthesis [5]. In adults, the acquisition of creatine via the diet or de novo synthesis is purported to be 50:50 [5]. However, due to low levels of creatine in human breast milk and commercial formulas, a term baby most likely synthesizes 64–93% of their daily creatine requirements [132]. When the human fetus/newborn develops the capacity to synthesize creatine is unknown, but it requires sufficient renal, pancreas and hepatic maturity to express the enzymes necessary for creatine synthesis [132]. Studies conducted in the precocial spiny mouse suggest that preterm infants are unlikely able to synthesize creatine before an age equivalent to ~35 weeks’ gestation, due to the developmental immaturity of kidney and liver limiting production of the AGAT and GAMT enzymes [133]. These observations around dietary creatine intake and endogenous synthesis raise questions about whether creatine insufficiency occurs in infants born preterm (before 37 completed weeks of gestation). This is important as the global incidence of preterm birth is reported to be ~11% [134] with rates stable or increasing in many parts of the world. Children and adults born preterm, even those discharged from the neonatal intensive care unit free of any gross cerebral injury have an increased risk of developing neurological disorders that mirror many of those experienced by CDS patients, including impaired executive function, developmental delay, psychiatric and behavioral sequelae [134]. Crucially too, the more preterm the birth, the greater the risk of later neurodevelopmental problems [135]. Finally, maternal mental health is also a powerful modulator of preterm birth outcomes. Psychological distress in otherwise healthy women is known to increase rates of preterm birth, with emergent data also suggesting that the fetuses of these women have altered brain development and reduced cerebral creatine concentrations [136]. Infants born preterm to mothers with mental health difficulties may therefore be at heightened risk for later neurodevelopmental problems.

There have been several small observational studies that have identified perturbations in creatine homeostasis in preterm infants [127,137]. A study by Koob et al. (2016) reported reduced creatine concentrations in the centrum semiovale of preterm infants (born 29.1 ± 2 weeks) when compared to term controls at term-corrected age [137]. When going on to consider systemic creatine levels, a study completed by Lage et al. (2013) found that by the time of hospital discharge, preterm infants had higher urinary GAA and reduced urinary creatine excretion. This was particularly apparent in their very preterm group (28–29 weeks) [138]. The role of systemic creatine homeostasis on cerebral creatine levels has not been adequately evaluated in preterm infants, but this should be explored further, as an inability to methylate GAA to produce creatine could be detrimental to the preterm infant, not merely because of creatine depletion, but also because increased levels of GAA can be neurotoxic [129]. With improvements in perinatal care and lowering gestational age threshold of human viability, the population of children and adults born <28 weeks’ gestation will increase. Key periods of brain development, usually occurring during the third trimester of pregnancy, need to be supported in an ex utero environment where there is no longer a custom pipeline of placental/maternally derived nutrients, including creatine. At these gestational ages, nutrition is largely supported by intravenous (parenteral) nutrition, which is creatine deplete until the gut is functionally mature enough to tolerate milk feeds, which are also creatine deficient. Parenteral nutrition is also associated with hepatotoxicity and neonatal cholestasis is a well-recognized complication; whether this further impairs the preterm infant’s ability for endogenous creatine synthesis is unknown.

No studies to date have established whether preterm infants develop cerebral creatine deficiency or whether a reduction in cerebral creatine content is associated with neurodevelopmental outcomes. Further to this, no study has monitored systemic creatine levels (both circulating and excreted) in a single preterm population, nor have they assessed nutritional creatine availability in total parenteral nutrition, preterm infant formulas or preterm maternal or donor breastmilk. These are the aims of the Understanding Creatine for Neurological Health (UNICORN) in babies observational cohort study currently underway [139]. The anticipated findings of this study are that low levels of creatine will be detected in preterm breastmilk and infant formula, placing a large burden of de novo creatine synthesis on the preterm infant. It is hypothesized that these babies will not be able to sustain creatine accretion; thus, ^1^H-MRS examination will show that preterm infants have lower cerebral creatine concentrations compared to those born at term. Whether or not creatine deficiency is associated with neurological deficit will be a secondary outcome of this study. The authors contend that detection and measurement of cerebral creatine perturbations in the preterm infant could provide the basis of early intervention with dietary creatine.

## 7. Conclusions and Research Road Forward

It is clear that creatine is involved in energy metabolism throughout female reproduction. Specifically, this review highlights the potential importance of creatine metabolism for successful fertilization. While many unanswered questions remain, there is clear evidence that both the endometrium and myometrium can use the creatine kinase circuit for energy homeostasis with indications that adaptations to creatine metabolism occur across the uterine reproductive cycle, and during pregnancy and parturition. What remains to be established is the functional importance of creatine metabolism within the various layers of the human uterus during the menstrual cycle and in the pregnant state. Overall, whether creatine deficiencies can be linked to sub-optimal fertility in females, as well as the capacity to use dietary creatine supplements to improve reproductive outcomes, warrants further investigation. This should be considered for both natural pregnancies and within the realm of artificial reproductive therapies. Indeed, the potential utility of creatine in IVF media should not be overlooked.

A surprising development in recent years has been the degree by which maternal creatine metabolism shifts with pregnancy; in particular, the capacity of the human placenta to synthesize creatine and that these processes are disturbed in pregnancy complications where oxygen, and thus cellular energy, depletion underpin pathology. To advance this research further, there is a need to uncover the mechanisms driving these changes. Specifically, when in gestation energy collapse may be imminent, and which pregnancies may benefit from creatine supplementation to safeguard against subsequent placental dysfunction and fetal compromise. The required increases in maternal creatine concentrations during pregnancy also raise questions about the use of dietary creatine supplements in settings where access to adequate nutrition, particularly animal protein, is limited.

Our understanding of the potential use of dietary creatine supplementation during pregnancy to improve outcomes for the neonate following intrapartum complications is further advanced. The potential use of maternal dietary creatine supplementation during pregnancy as a prophylactic treatment for fetal hypoxia and perinatal brain injury is exciting, as this treatment may prove beneficial in all resource settings globally. Further to this, observational studies underway in preterm infants will soon inform the medical community on whether the simple inclusion of creatine in preterm nutrition may support ex utero brain development and function, ultimately reducing the risk of long-term neurological deficit in these vulnerable babies. Translation of these pre-clinical in vitro, animal and human observational studies are still on the horizon. While it may appear simple to implement the use of an already available nutritional supplement in pregnancy, before initiating clinical trials, we must always consider the consumer perspective. The recently conducted Acceptability of Dietary or Nutritional Supplements in pregnancy (the ADONS Study), explored knowledge of, and acceptance of, introducing creatine as a nutritional supplement in late pregnancy [140]. This study assessed the perspectives of pregnant women, their families and healthcare providers, concluding that creatine would be an acceptable supplement during pregnancy provided they were given evidence-based assurances of efficacy and safety. There is no indication that creatine supplements produced under high-quality manufacturing standards and consumed following manufacturer’s directions pose any safety risks or cause adverse side-effects in women of reproductive age or preterm infants [141,142]. However specialized safety and tolerability studies in pregnant women or those trying to conceive are still required. Overall, the available literature supports creatine metabolism being considered an essential component of bioenergetics for successful reproduction, and one may be cautiously optimistic, with further research, about the potential impact of creatine supplementation to improve reproductive and perinatal outcomes.

## Figures and Tables

**Table 1:** Summary of creatine metabolism in the female reproductive tract.

Tissue	Species	Creatine and Phosphocreatine Content	Creatine Kinases	Creatine Synthesis and Transport
Oocytes	Mouse	Creatine and phosphocreatine present (~4 to 5 mmol.kg^−1^ dry mass) [16].	CKBB gene, protein and activity reported [16,17]. Activity increased with oocyte maturation and fertilization [18]. *CKMM* detected. Expression levels increased with hCG stimulation [9].	
	Bovine		uMt-CK and CKBB gene and protein expression reported [18]. Use of CK inhibitors elevated intra-oocyte ADP:ATP ratio [19].	
	Human	Creatine and phosphocreatine present (~4 to 5 mmol.kg^−1^ dry mass) [16].		
Ovaries	Rat			High *SLC6A8* gene expression reported [21].
Ovarian stromal cells	Human			Detectable levels of the *GATM* gene and AGAT protein, but *GAMT* undetected [22].
Cumulus cells or cumulus–oocyte complexes (COCs)	Human		*CKBB* gene expression detected and elevated in women with good quality embryos undergoing ART [23].	
	Bovine	Creatine and GAA detected in media bathing cells, with an increase in creatine (~450-fold) and GAA (~2-fold) reported during in vitro maturation [24].		
Follicular fluid	Human	Creatine detected and lower in women with endometrioma [30].		
	Mouse	Creatine detected and increases around ovulation [9].		
	Equine	Creatine detected. Remains unchanged with follicular development [31].		
Granulosa cells	Rat			Increase in *GATM* and *GAMT* expression with equine CG stimulation [9].
Oviduct	Human			*GATM* and *GAMT* and *SLC6A8* detected [21,22]
	Rat			*GATM* and *GAMT* and *SLC6A8* detected [21,22]
	Mouse			*GAMT* gene and protein not expressed [32]
Oviductal fluid	Equine	High creatine concentration (3–4 mM) that did not change pre- to post-ovulation [33,34].		
	Mouse	Creatine levels detected and increased with hCG stimulation [9].		*GATM* and *GAMT* detected. No change in expression with hCG stimulation [9].
Non-pregnant Endometrium	Human		Up-regulation of *CKBB* expression and enzyme activity in the secretory phase of the menstrual cycle [37,39,40,41].	Increased *SLC6A8* expression during the secretory phase of the menstrual cycle [37].
Pregnant endometrium	Rat		uMt-CK and CKBB proteins expressed in the decidua parietalis and basalis [36].	AGAT activity high in the decidua. No GAMT enzyme activity present [48].
	Sheep			GAA produced at a higher level than non-pregnant animals [49].
	Human		Creatine kinase activity present in term decidual explants [46].	
Non-pregnant myometrium	Human	Phosphocreatine detected at a low level compared pregnant myometrium [51].	Creatine kinase activity detected [50].	
Pregnant myometrium	Human	Phosphocreatine detected with higher levels at term compared to non-pregnant tissue [55].		*CKBB* gene expression detected. Levels were three-fold higher at term compared with earlier in gestation [53].

Abbreviations—cytosolic brain-type creatine kinase (CKBB), cytosolic muscle-type creatine kinase (CKMM), ubiquitous mitochondrial creatine (uMt-CK), guanidinoacetate (GAA), artificial reproductive therapy (ART), human chorionic gonadotropin (hCG), chorionic gonadotropin (CG).

**Table 2:** Summary of Creatine Metabolism in the Human Placenta.

Study	Condition	Gestation	Creatine and Phosphocreatine Content	Creatine Kinases	Creatine Synthesis and Transport
Thomure et al. [64]	Healthy	First, second and third trimester		*uMt-CK* and *CKBB* gene expression detected. Expression was low in the first and second trimester before a peak at term. CKBB protein expression consistent throughout gestation. uMt-CK expression rose through to mid-gestation before declining just before term.	
Ellery et al. [13]	Healthy	First trimester (10–13 weeks’ gestation)			*GATM*, *GAMT* and *SLC6A8* detected.
Ellery et al. [13,65]	Healthy	Third trimester			AGAT, GAMT and SLC6A8 gene and protein detected. *GATM* expression and GAA tissue content decreased with advancing gestational age and birth weight.
Tissot et al. [69]	High altitude	Term	Increased phosphocreatine levels detected.		
Ellery et al. [13]	FGR	Third trimester	43% higher total creatine content compared to gestation-matched controls.		2-fold increase in *SLC6A8* expression.
Ellery et al. [14]	PE	Third trimester	38% higher total creatine content compared to gestation-matched controls.	Increased *CKBB* mRNA expression.	Increased *GATM*, *GAMT*, *SLC6A8* mRNA expression.
Jääskeläinen et al. [71]	PE	Term	Increase in creatine concentration in venous cord plasma at delivery.		
McMinn et al. [74]	FGR	Term			Down-regulation in *GATM*.

Abbreviations—fetal growth restriction (FGR), preeclampsia (PE), cytosolic brain-type creatine kinase (CKBB), ubiquitous mitochondrial creatine (uMt-CK), the creatine transporter (SLC6A8), guanidinoacetate (GAA), arginine:glycine amidinotransferase (AGAT translated from the *GATM* gene), guanidinoacetate methyltransferase (GAMT).

**Table 3:** Creatine Treatment in Animal Models of Perinatal Brain Injury.

Species	Developmental Timing	Treatment	Main Outcomes
Guinea pigs and Rats [113]	Fetal guinea pigs (0.9 gestation) Or neonatal rats (P7)	2 h creatine treatment to hippocampal slices in vitro or injection of 3g/kg creatine before and after hypoxic-ischemic insult.	Creatine improved recovery of brain protein synthesis, reduced infarction and neuronal cell injury.
Mice [114]	Neonatal (P0–5) and juvenile (P6–13)	Maternal dietary creatine supplementation (2 g/kg/day) or incubation of brain slices (200 μM) creatine.	Creatine preserved ATP turnover and reduced neuronal injury.
Rat [115]	Neonatal or juvenile (P10–15)	Subcutaneous creatine (3 mg/g of body weight) for 3 days before hypoxic insult.	Low phosphocreatine/creatine ratio led to higher susceptibility of seizures. Creatine improved survival and prevented seizure activity.
Rabbit [116]	5 to 30 day-old pups	Subcutaneous creatine (3 mg/g of body weight) for 3 days before hypoxic insult.	Creatine increased brain PCr/NTP ratio and prevented hypoxic seizures.
Rat [117]	Neonatal (P6)	Subcutaneous creatine (3 g/kg body weight/day) for 3 days before hypoxic insult.	Creatine prevented brain oedema associated with severe hypoxia-ischemia.
Spiny Mouse [118]	Fetal (term) and juvenile (P15)	Maternal dietary creatine supplementation (5% *w*/*w* from mid-gestation).	Creatine increased pup survival and improved postnatal growth.
Spiny Mouse [118]	Neonatal (P1)	Maternal dietary creatine supplementation (5% *w*/*w* from mid-gestation).	Creatine reduced perinatal mortality and pro-apoptotic protein BAX, cytoplasmic cytochrome *c*, and caspase-3 in the fetal brain.
Spiny Mouse [119,120,121,122]	Neonatal (P1) or juvenile (P35)	Maternal dietary creatine supplementation (5% *w*/*w* from mid-gestation).	Creatine prevented structural and functional damage to the diaphragm [119,120], skeletal muscle [121], and kidney [122].
Spiny Mouse [123]	Adult (P90)	Maternal dietary creatine supplementation (5% *w*/*w* from mid-gestation).	Creatine decreased the risk of male offspring developing chronic kidney disease.

Abbreviations—postnatal age (P), phosphocreatine/nucleoside triphosphate ratio (PCr/NTP), weight/weight (*w*/*w*).

## Data Availability

Not applicable.
